# A randomised non-inferiority controlled trial of a single versus a four intradermal sterile water injection technique for relief of continuous lower back pain during labour

**DOI:** 10.1186/1471-2393-11-21

**Published:** 2011-03-23

**Authors:** Nigel Lee, Peter Coxeter, Michael Beckmann, Joan Webster, Vanessa Wright, Tric Smith, Sue Kildea

**Affiliations:** 1 Mater Health Services, South Brisbane, Qld. 4101 Australia; 2 Mater Medical Research Institute, South Brisbane, Qld. 4101 Australia; 3 Royal Brisbane and Women's Hospital, Herston, Qld. 4029 Australia; 4 Australian Catholic University, Banyo, Qld. 4014 Australia

## Abstract

**Background:**

Almost one third of women suffer continuous lower back pain during labour. Evidence from three systematic reviews demonstrates that sterile water injections (SWI) provide statistically and clinically significant pain relief in women experiencing continuous lower back pain during labour. The most effective technique to administer SWI is yet to be determined. Therefore, the aim of this study is to determine if the single injection SWI technique is no less effective than the routinely used four injection SWI method in reducing continuous lower back pain during labour.

**Methods/design:**

The trial protocol was developed in consultation with an interdisciplinary team of clinical researchers. We aim to recruit 319 women presenting at term, seeking analgesia for continuous severe lower back pain during labour. Participants will be recruited from two major maternity hospitals in Australia. Randomised participants are allocated to receive a four or single intradermal needle SWI technique. The primary outcome is the change in self-reported pain measured by visual analogue scale at baseline and thirty minutes post intervention. Secondary outcomes include VAS change scores at 10, 60, 90 and 120 min, analgesia use, mode of birth and maternal satisfaction.

**Statistical analysis:**

Sample size was calculated to achieve 90% power at an alpha of 0.025 to detect a non-inferiority margin of ≤ 1 cm on the VAS, using a one-sided, two-sample t-test. Baseline demographic and clinical characteristics will be analysed for comparability between groups. Differences in primary (VAS pain score) and secondary outcomes between groups will be analysed by intention to treat and per protocol analysis using Student's t-test and ANOVA.

**Conclusion:**

This study will determine if a single intradermal SWI technique is no less effective than the routinely used four injection technique for lower back pain during labour. The findings will allow midwives to offer women requesting SWI during labour an evidence-based alternative technique more easily administered by staff and accepted by labouring women.

**Trial Registration:**

ACTRN12609000964213

## Background

Almost one in three (30%) women in labour suffer from continuous lower back pain [1]. This pain is often associated with varying degrees of fetal malposition, particularly occipito-posterior position, which may apply pressure on pain-sensitive structures within the pelvis [2]. Characteristically, the pain persists throughout the normal painless resting intervals between contractions and is associated with greater analgesic requirement [2].

Administration of Sterile Water Injections (SWI) into the lower back is used in midwifery to provide pain relief to women experiencing lower back pain during labour. The sterile water causes osmotic and mechanical irritation resulting in a brief (15-30 second) and significant stinging sensation. The onset of pain relief follows almost immediately and may last for up to two hours. The procedure can be repeated a number of times [3]. The therapeutic effect has been explained by gate control theory [4] whereby the painful stinging stimulates competing nerve fibres, creating a block to the slower visceral signals from uterine contractions and back pain. The most frequently used SWI technique consists of four intradermal injections into the skin surrounding the Michaelis rhomboid over the sacral area.

Systematic reviews [3,5] and meta-analysis [6] have reported a significant reduction in self-reported pain measures in groups receiving SWI compared with controls. Authors concluded that SWI are an effective therapeutic intervention for the management of continuous back pain during labour. However, the included randomised controlled trials (RCT) were heterogeneous and incorporated different SWI techniques (single and four-injection SWI methods), methods of administration (intradermal or subcutaneous), and comparison groups (normal saline, transcutaneous electronic nerve stimulation and "standard care" defined as massage, counter pressure and water immersion). Authors of the systematic reviews [3,5] highlight that the analgesic efficacy obtained following administration of a single SWI at the most painful point [7] appear comparable to that obtained using the four injection SWI technique. The analgesic benefit of the single SWI method is further supported by results of a more recent randomised controlled trial (RCT) [8] which compared a single SWI to a placebo. The comparable analgesic benefit observed for both techniques has led authors to conclude there is need for further research [3,5]. There have been no trials conducted to date comparing the two SWI techniques.

There may be important clinical benefits in demonstrating the comparable analgesic efficacy of a single versus four needle SWI technique. The relative reduction in discomfort associated with the single injection procedure may increase the woman's satisfaction and acceptability with SWI treatment during labour, and willingness to use the procedure again in the future. In addition, improved resource allocation can be expected as only one midwife is required to administer a single SWI compared with the current recommendation for two midwives to administer the four injection technique. Therefore, the aim of this study is to compare a single intradermal SWI technique with the standard four injection intradermal technique in the degree and duration of analgesic benefit.

## Methods/design

A randomised, controlled, non-inferiority design was considered most appropriate method to answer our stated aim. Where two treatments, have both previously been shown to be superior to a placebo, and/or in cases where the use of a placebo may be unethical or impractical, a non inferiority design can be used to determine if one treatment is "no worse" than the other[9,10]. Usually, as in this study, the interventions include a new treatment and an active comparator, or a treatment currently in use. In relation to this trial, the single injection is the new treatment and the four injection technique is the active comparator. The null hypothesis is not proven if the measured effects of the two treatments lie within a specified non-inferiority margin [11].

### Hypothesis

The null hypothesis is conventionally stated in an RCT which assumes that there is no difference between the intervention group and controls. The null hypothesis places the onus on the intervention to be proven [12]. The null hypothesis is accepted if it cannot be refuted at the *a priori *defined level of significance. However, a non-inferiority design is attempting to show that two active treatments are similar in effect [13]. Therefore, the null hypothesis cannot be rejected if the measured treatment effects lie within a pre-specified non-inferiority margin [11]. Within non-inferiority design the sample size must be robust enough to test the quantitative or clinically relevant margin or definition of non-inferiority [13]. This margin is informed by considerations of clinically relevance, with any treatment differences falling outside this range indicating that any dissimilarity between treatments is unacceptably large [9].

The predefined non-inferiority margin for the present study was defined by the minimal clinically (i.e. not statistically) significant difference in the Visual Analogue Scale (VAS) to measure self-reported pain. Therefore, the null hypothesis (H_0_) would be "treatment is inferior", the alternative hypothesis (H_A_) "treatment is non-inferior":

H_0_: Difference between the four injection intradermal SWI and single injection intradermal SWI is ≥ 1 cm on the 10 cm VAS

H_A_: Difference between the four injection intradermal SWI and single injection intradermal SWI is < 1 cm on the 10 cm VAS

### Study site and population

The study population will be recruited from the Birth Suites of two major maternity hospitals. Women eligible for the study are those that request analgesia for continuous severe lower back pain during labour (≥7 on the VAS) and provide informed consent. The categorisation of severe back pain as ≥7 on the VAS has been previously validated [14] and consistent with defined inclusion criteria in previous SWI studies [14-17]. Women with a baseline VAS score of <7 were more likely to find administration of the procedure unacceptable [16].

The eligibility criteria are pre-specified as:

### Inclusion criteria

Participants eligible for the study will be defined as:

Women at term (between 37 and 42 weeks)

Nulliparous or multiparous

Singleton pregnancy

Cephalic presentation

First stage labour (spontaneous or induced)

No previous analgesia pharmacological analgesia (nitrous oxide inhalation, narcotics)

Back pain assessed by VAS as ≥7

Ability to give informed consent. This may exclude women of non-English speaking backgrounds, where an interpreter is unavailable, and those women whose consent is required to be provided by a parent or guardian.

### Exclusion criteria

Gestation <37 weeks

Multiple pregnancy

Malpresentation (Breech Transverse etc.)

Second stage labour

Pharmacological analgesia prior to SWI

Back pain assessed by VAS <7

Women whose labour would be considered high risk.

### Interventions

#### Control group

**Figure 1:**
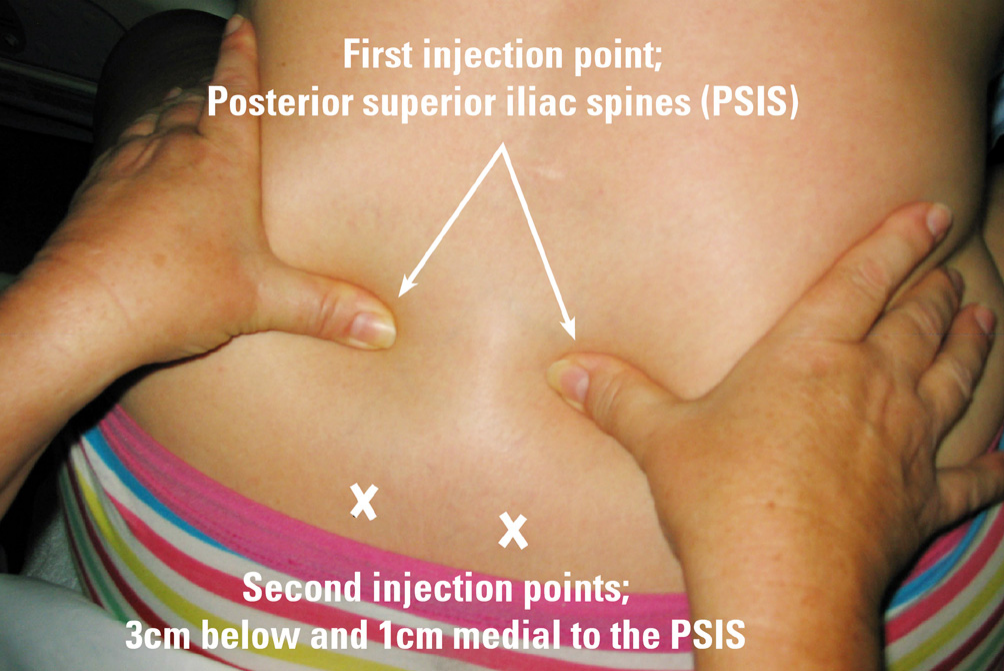
**The four injection technique**.

Participants randomised to the control group will be given SWI using the standard four injection intradermal technique into the skin surrounding the Michaelis rhomboid over the sacral area. Anatomically, the injections will be administered: two over each posterior superior iliac spine (PSIS) and two three cm below and one cm medial to the PSIS (Figure 1). Minor discrepancies or changes to the anatomical position or alignment of the four injections have not been shown to impact on any analgesic effect [18].

#### Intervention group

Participants randomised to the intervention group will be given SWI using the single injection intradermal technique.

**Figure 2:**
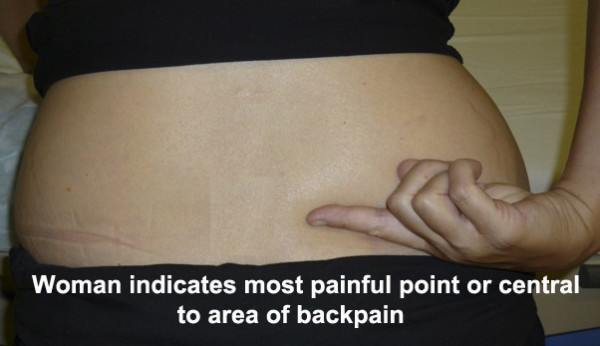
**The single injection technique**. Woman indicates the most painful point or central to the most painful area.

The anatomical site for the single injection will be over the single most painful point as indicated by the woman (Figure 2).

#### Primary outcome

Decrease in pain measured by VAS at 30 minutes post-intervention.

#### Secondary outcomes

Secondary outcomes will include:

a) Pain score measured by VAS 10, 60, 90 and 120 mins post-intervention

b) Level of administration discomfort associated with SWI procedure (measured by VAS at 10 minutes post SWI)

c) Subsequent analgesia use (pharmacological and non-pharmacological)

d) Mode of birth

e) Likelihood to use again with subsequent labour

f) Patient satisfaction with analgesic effect

### Sample size

A sample size of 133 in each group will be required to achieve 90% power to detect non-inferiority using a one-sided, two-sample t-test. The significance (alpha) of the test is 0.025. The non-inferiority margin is ≤ 1 cm on the VAS and the true difference between the means is assumed to be zero (cms). A standard deviation (SD) of ± 2.5 on the VAS was conservatively estimated from results reported in a recent meta-analysis [6] of SWI studies. As consent and randomisation will occur in the birth suite, attrition is expected to be minimal. However, we have estimated a 20% attrition to account for cases of precipitate birth prior to the assessment of the primary outcome, or other unforseen reasons for withdrawal such as emergency caesarean section. Thus a total of 319 participants will be required.

### Recruitment of participants

Trial participation is invited through public antenatal clinics, antenatal education classes and on the Birth Suites at both recruiting hospitals. Although both sites use a number of models of care in the provision of maternity services, all women return to the public antenatal clinic at a particular gestation; 36 weeks and 30 weeks at the Site One and Site Two, respectively. At this time women will be provided with information regarding the trial. The recruitment strategy aims to provide information regarding the trial prior to presentation to the Birth Suite in labour.

### Consent

The recruitment strategies are designed to ensure that women receive information regarding SWI as an intervention for back pain and information on the trial, at or before 36 weeks gestation (i.e. prior to inclusion criteria of 37-42 weeks gestation). The majority of women will consent to the trial on presentation to the Birth Suite in labour at both sites. Clinical midwives and/or clinical facilitators from both sites will be available to receive informed consent. A trial investigator will be available via mobile phone to provide support for midwives undertaking consent and enrolment procedures, and to promote continuity of procedures and trial fidelity across the two sites. Women who have been provided with a participant information sheet and indicate an interest in joining the trial will be able to contact the research midwife or investigators for further information or clarification before consent. A video demonstrating the four injection technique is available for women to access at the trial website. http://maternity.mater.org.au/switch. A form will be placed with the woman's chart to highlight that the woman has consented to participate in the study if eligibility criteria is met. Verbal consent will be re-affirmed immediately prior to randomisation and documented within in the woman's clinical chart. Women are able to withdraw consent at any time (i.e. before, during or after the procedure and/or before completing follow-up questionnaire) without affecting their usual clinical care or request alternative and available means of analgesia.

### Randomisation

**Figure 3:**
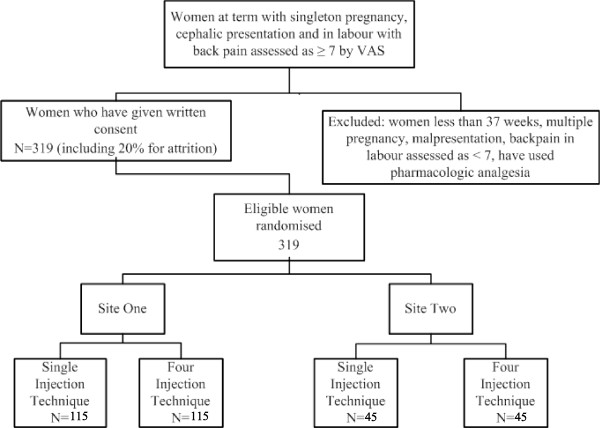
**Randomisation and participant flow**.

The randomisation schedule for Site One will be prepared by a statistician using a computer-generated list of random numbers. Blinding of allocation to the study intervention will be undertaken using opaque sealed envelopes prepared by Site One administrative staff. A separate randomisation sequence was generated for Site Two in permuted blocks of four to ensure an equal number of participants across both arms of the trial where smaller recruitment numbers are expected. Blinded allocation at this site will be undertaken using similarly prepared opaque sealed envelopes (Figure 3).

### Blinding

At both sites, the envelopes will be kept in a locked cupboard on the birth suite and available for randomisation 24 hours/day. The key will be held by the Midwifery Team Leader who will obtain and open one envelope, then with the assistance of another midwife will administer the SWI using the technique detailed within the envelope. The two midwives will be present during the procedure regardless of the SWI technique being administered, to assist with blinding of the procedure to the midwife providing care and collecting data. The injection site(s) will be covered with a hypoallergenic opaque dressing to prevent visualisation of the number of injections used. The participants will be aware not to discuss the type of intervention they were allocated to with their midwife. The midwife caring for the woman will then return to collect the intervention data.

### Data collection

Upon randomisation the attending Midwife will document the following data:

• UR number

• Gravity and Parity

• Gestation

• Spontaneous or induced labour

• Findings of last vaginal exam and time

• VAS score and time

• Time of administration

Following administration of SWI the following data will be recorded:

• VAS related to analgesia effect at 10 mins

• VAS related to discomfort felt during the procedure (at 10 mins)

• VAS related to analgesic effect at 30 minute intervals up to 2 hours post procedure

At Site One the following data will be extracted from the obstetric database by the research midwife following birth:

• Age

• Booking weight & BMI

• Mode of birth

• Time of birth

• Any other analgesia used (pharmacological and non pharmacological)

• Model of care

• Educational level

The obstetric databases at Site One and Site Two vary slightly in the data items collected. As separate data collection forms have been generated for the randomisation process for Site Two, the data collection forms have been amended to account for any differences in information captured by the database.

The following self-reported data will be sought from the woman within 48 hours following birth:

• Perceived satisfaction with SWI administration

• Perceived satisfaction with SWI analgesic benefit

• Likelihood to use again with subsequent labour

• Likelihood to recommend to SWI to others

Data will be collected by the Midwife caring for the labouring woman then returned to a securely locked cupboard. Completed data forms will be de-identified and entered into a password protected electronic database. Identified data collection forms will be kept in locked storage for 15 years. Data collection forms at the second site will be stored in a locked cupboard and collected on a fortnightly basis. Data will be entered into a Microsoft Excel spreadsheet and transmitted as a password protected file

### Data collection tools

The intrapartum data collection tool has been adapted from an existing document that was used as an audit tool during the introduction of SWI at Site One. For self reported postnatal data, the data collection tool previously published and used in the Australian study by Peart et al. [16] has been made available for use. This tool has been tested for face readability, language and face validity. The instrument will be administered by a researcher blinded to the particular technique used.

### Statistical analysis

**Figure 4:**
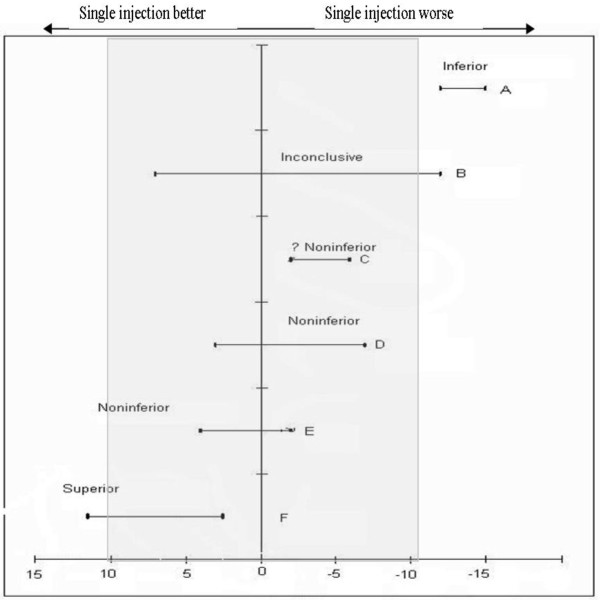
**Possible scenarios of observed treatment differences [adapted from **[22]**]**. Shaded area indicates non-inferiority margin.

Demographic and other baseline characteristics will be analysed for comparability between groups to assess the adequacy of randomisation. Differences in VAS pain score between the two groups will be analysed using Student's t-test and analysis of variance (non-parametric tests used if data is highly clustered toward extremes). Data analysis for the primary outcome will determine if the treatment effect lies within the a priori defined non-inferiority margin (± 1 cm) and the null effect (0 cm) at a one-sided alpha of 0.025. The pre-specified non-inferiority margin was based on a one cm (95% CI 0.6-1.4 cm) minimally clinically significant difference reported for VAS in severe pain [14]. The Mann-Whitney U test will be utilised for non-parametric data and chi-squared test for categorical variables. Women who give birth or elect for narcotic and/or regional analgesia after the collection of the primary measure (VAS score 30 minutes post injection) but before the completion of the secondary outcomes (VAS up to 120 mins) will be included in the trial. Women giving birth or electing for narcotic and/or regional anaesthesia prior to the collection of the primary outcome measure will be included in the Intention to Treat (ITT) analysis. The conservative nature of ITT may obscure differences between treatments groups therefore increasing the chance of falsely declaring equivalence [19]. To address this, some authors have recommended that Per Protocol (PP) analysis also be conducted alongside ITT to provide a more robust conclusion if both methods support the non-inferiority [9,20]. Other authors [21] argue that the benefits of ITT analysis continue to outweigh the PP approach. As it is unlikely that participants in this trial will cross from one intervention to another, the ITT approach will be used unless the dropout out rate exceeds the estimated margin allowed of 20%. Should the dropout rate exceed 20% then a PP analysis will also be conducted. Figure 4 outlines possible scenarios for the outcome of the data analysis based on the pre-defined noninferiority margin, indicated as the shaded area. Error bars indicate 90% confidence intervals (CI). If the CI is completely to the left of zero, the treatment is inferior (scenario A). Scenarios C,D and E indicate noninferiority as the CI lies within the specified margin (shaded area). Scenario F would indicate superiority. An inconclusive result (scenario B) is unlikely as the sample size is sufficiently powered to provide a result [22].

In the event that women experience no relief from the single injection and request the four injection technique, these women will remain within the study, and the data will be analysed according to ITT and per protocol principles.

Women requesting a repeat injection will receive the standard four injection technique. A pre-injection VAS score will be sought prior to administration and post injection VAS score will be collected at 30 minutes. These will be reported separately.

### Confidentiality and data security

The data is de-identified, coded and entered onto a password protected database.

### Data safety monitoring board

A Data Safety Monitoring Board has not been established for this trial. As there have been no adverse events reported in any previous trial on the topic, the trial was considered to be low risk. However, a senior obstetrician at both sites has agreed to act as a clinical monitor to investigate any adverse events that may be associated with the trial. This process has been approved by the Human Research Ethics Committee at each site.

### Project management

Project management will be overseen by the research team that consists of midwifery and obstetric clinicians, midwifery academics and researchers from both sites. The research team will meet regularly during the projects development phase and regular contact and progress reports maintained via email during the recruitment phase of the trial.

### Ethical issues

Human Research Ethics Committee (HREC) Approval

Mater Health Services HREC   No:1422M

Australian Catholic University HREC   No:Q2009 48

Royal Brisbane and Women's Hospital HREC   No:HREC/10/QRBW/155

#### Women of Non-English Speaking Backgrounds (NESB) and minors

At both sites, an onsite interpreter service is available during "office hours" for the most common language groups of Vietnamese and Mandarin/Cantonese with contract interpreters for eighteen other languages available. At other times the Birth Suite accesses a telephone interpreter service. For NESB women considering SWI as an intervention for back pain, the interpreter service accessed at the time will be utilised to provide information and gain consent for inclusion in the trial. To supplement this, copies of the Participant Information Sheet and consent form will be available in Vietnamese and Mandarin/Cantonese. In the event that a suitable interpreter cannot be found, inclusion in the trial will not be offered. Postnatal data will be collected using this method as interpreters are required when providing routine postnatal information.

#### Potential risks

The four injection SWI technique is routinely used in the birth suites at Site One. Therefore, no additional risks from study participation would be expected. Moreover, no allergic or systemic reactions to the procedure have been reported in the literature other than the brief stinging sensation immediately following administration [5].

## Discussion

This study employs a randomised controlled non-inferiority design determining if the single SWI technique is non-inferior to the four SWI technique more commonly used in clinical practice. This research gap was identified in the literature.

The project has the potential to make a significant contribution to maternity and midwifery research and patient care. Nationally and internationally, maternity care is offered in a diverse range of settings and models of care, offering different services and options in terms of pain management. Many women may not have access to narcotics and epidural analgesia, or these forms of analegisia may be unacceptable due to actual or perceived side effects or cultural considerations. The implementation of a best practice, evidenced based SWI technique for the relief of back pain in labour will provide an alternative analgesic option that addresses consumer demand for low intervention strategies. The procedure is not technology dependent, relatively simple, and may be an effective and feasible analgesic strategy suitable for any maternity care setting or model. Therefore, this trial will make a significant contribution to the evidence supporting the most effective administration of SWI. Important clinical (e.g. increased women's satisfaction/acceptability of SWI administration) and cost benefits (e.g. reduced staff/time required for administration) might be expected if the analgesic efficacy of the single needle technique can be shown to be no less effective than the four injection method.

## Competing interests

The authors declare that they have no competing interests.

## Authors' contributions

NL originally conceived the study and has overall responsibility for the trial; NL and PC designed the study and drafted the initial study protocol; SK, MB and JW reviewed the study protocol; NL wrote the grant applications which were reviewed by PC and SK: NL developed the data collection tools and processors; VW and PS co-ordinate recruitment; NL with the assistance of PC, JW and SK will undertake the data analysis; NL, PC, SK, JW and MB drafted the trial protocol manuscript. All authors read and approved the final manuscript.

### Project team

#### Principal investigator

Nigel Lee (Australian Catholic University/Mater Health Services)

#### Associate investigators

Prof. Sue Kildea (Australian Catholic University/Mater Health Services)

Peter Coxeter (Mater Medical Research Institute)

Vanessa Wright (Mater Health Services)

Dr Michael Beckmann (Mater Health Services)

Adj.Prof. Joan Webster (Royal Brisbane and Women's Hospital)

Patricia Smith (Royal Brisbane and Women's Hospital)

#### Research midwives

Rebecca Cavallaro (Mater Medical Research Institute)

Louise O'Beirne (Royal Brisbane and Women's Hospital)

## Funding

Funding for the trial has been received from

The John P Kelly Research Foundation

Queensland Nursing Council Research Funds

Assoc. Prof. Joan Webster

Mater Foundation

Golden Casket Research Scholarships

## Pre-publication history

The pre-publication history for this paper can be accessed here:

http://www.biomedcentral.com/1471-2393/11/21/prepub

## References

[B1] Melzack R Schaffeberg D Low-back pain during labour American Journal of Obstetrics and Gynecology 1987 158 901 905 10.1016/0002-9378(87)90349-8 2953242

[B2] Simpkin PBA Update on Nonpharmacologic Approaches to Relieve Labor Pain and Prevent Suffering J Midwifery Womens Health 2004 49 6 489 450 15544978 10.1016/j.jmwh.2004.07.007

[B3] Martensson L Wallin G Sterile water injections as treatment for low-back pain during labour: a review Aust N Z J Obstet Gynaecol 2008 48 4 369 74 10.1111/j.1479-828X.2008.00856.x 18837842

[B4] Melzack R Wall P Pain mechanisms: a new theory Science 1965 150 699 971 979 10.1126/science.150.3699.971 5320816

[B5] Fogarty V Intradermal sterile water injections for the relief of low back pain in labour -- a systematic review of the literature Women & Birth 2008 21 4 157 163 10.1016/j.wombi.2008.08.003 18926789

[B6] Hutton EK Sterile water injection for labour pain: a systematic review and meta-analysis of randomised controlled trials BJOG: An International Journal of Obstetrics & Gynaecology 2009 116 9 1158 1166 10.1111/j.1471-0528.2009.02221.x 19459860

[B7] Bahasadri S Subcutaneous sterile water injection for labour pain: a randomised controlled trial Australian & New Zealand Journal of Obstetrics & Gynaecology 2006 46 2 102 106 10.1111/j.1479-828X.2006.00536.x 16638030

[B8] Kushtagi P Bhanu BT Effectiveness of subcutaneous injection of sterile water to the lower back for pain relief in labor Acta Obstet Gynecol Scand 2009 88 2 231 3 10.1080/00016340802635534 19096945

[B9] Miller S Neate C Wang D Wang D, Bakhai A Noninferiority Trials Clinical Trials: A practical guide to design, analysis and reporting 2006 Remedica: London 131 140

[B10] Scott IA Non-inferiority trials: determining whether alternative treatments are good enough Medical Journal of Australia 2009 190 6 326 330 19296815 10.5694/j.1326-5377.2009.tb02425.x

[B11] Kaul S Good enough: A primer on the analysis and interpretation of noninferiority trials Annals of Internal Medicine 2006 145 1 62 69 16818930 10.7326/0003-4819-145-1-200607040-00011

[B12] Cluett E Cluett E, Bluff R Experimental Reseach, in Principles and practice of research in midwifery 2006 Curchill Livingston: Edinburgh

[B13] Piantadosi S Clinical trials: a methodologic perspective Wiley series in probability and statistics 2005 xxvii 2 Hoboken, N.J.: Wiley-Interscience 687

[B14] Collins S Moore R McQuay H The visual analogue pain intensity scale: What is moderate pain in millimetres Pain 1997 72 95 97 10.1016/S0304-3959(97)00005-5 9272792

[B15] Kelly A-M The minimum clinically significant difference in visual analogue scale pain score does not differ with severity of pain Emergency Medicine Journal 2001 18 3 205 207 10.1136/emj.18.3.205 11354213 PMC1725574

[B16] Peart K James W Deocampo J Use of sterile water injections to relieve back pain in labour Birth Issues 2006 15 1 18 22

[B17] Wiruchpongsanon P Relief of low back labor pain by using intracutaneous injections of sterile water: a randomized clinical trial J Med Assoc Thai 2006 89 5 571 6 16756038

[B18] Duff MM Sterile water injections for back pain in labour New Zealand College of Midwives Journal 2008 39 33(6)

[B19] Jones B Trials to assess equivalence: the importance of rigorous methods British Medical Journal 1996 313 7048 p36(4) 10.1136/bmj.313.7048.36 PMC2351444 8664772

[B20] D'Agostino RB Non-inferiority trials: design concepts and issues-the encounters of academic consultants in statistics Statistics in Medicine 2003 22 2 169 186 12520555 10.1002/sim.1425

[B21] Wiens BL The role of intention to treat in analysis of noninferiority studies Clinical Trials 2007 4 3 286 291 10.1177/1740774507079443 17715258

[B22] Piaggio G Reporting of Noninferiority and Equivalence Randomized Trials: An Extension of the CONSORT Statement JAMA 2006 295 10 1152 1160 10.1001/jama.295.10.1152 16522836

